# Gap Measurement of Point Machine Using Adaptive Wavelet Threshold and Mathematical Morphology

**DOI:** 10.3390/s16122006

**Published:** 2016-11-26

**Authors:** Tianhua Xu, Guang Wang, Haifeng Wang, Tangming Yuan, Zhiwang Zhong

**Affiliations:** 1 State Key Laboratory of Rail Traffic Control and Safety, Beijing Jiaotong University, Beijing 100044, China; guangwang@bjtu.edu.cn; 2 National Engineering Research Center of Rail Transportation Operation and Control System, Beijing Jiaotong University, Beijing 100044, China; hfwang@bjtu.edu.cn; 3 Department of Computer Science, University of York, York YO10 5GH, UK; tommy.yuan@york.ac.uk; 4 School of Electronic and Information Engineering, Beijing Jiaotong University, Beijing 100044, China; 12111056@bjtu.edu.cn

**Keywords:** edge detection, wavelet-based image denoising, image binarization, mathematical morphology

## Abstract

A point machine’s gap is an important indication of its healthy status. An edge detection algorithm is proposed to measure and calculate a point machine’s gap from the gap image captured by CCD plane arrays. This algorithm integrates adaptive wavelet-based image denoising, locally adaptive image binarization, and mathematical morphology technologies. The adaptive wavelet-based image denoising obtains not only an optimal denoising threshold, but also unblurred edges. Locally adaptive image binarization has the advantage of overcoming the local intensity variation in gap images. Mathematical morphology may suppress speckle spots caused by reflective metal surfaces in point machines. The subjective and objective evaluations of the proposed method are presented by using point machine gap images from a railway corporation in China. The performance between the proposed method and conventional edge detection methods has also been compared, and the result shows that the former outperforms the latter.

## 1. Introduction

The railway system in China has undergone a dramatic increase in recent years. According to a report of the National Railway Administration of the People’s Republic of China [1], the passenger and cargo transportation volume were 2.535 billion and 3.358 billion tons, respectively, in 2015. Heavy traffic means that the capacity utilization of the existing infrastructure is high. This will lead to more equipment failures and service disruptions. Among these equipment failures, railway point machines account for the vast majority of railway infrastructure failures that affect the availability of the system [2]. Almost 33% of the total maintenance cost of railways is dedicated to point machines and crossings [3]. How to monitor the health of railway turnouts and decrease their failure rates has become an important problem that urgently requires a solution.

**Figure 1:**
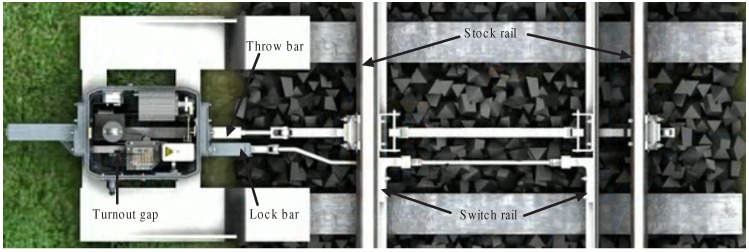
An example of a point structure.

The gap width between a switch point and a stock rail is a key safety parameter for monitoring a point machine’s healthy status. Too large of a gap may lead to catastrophic consequences, such as train derailment, human injury, and severe damages to the equipment and the environment [4]. Monitoring these parameters helps to build a point machine failure prediction system. Among these monitoring methods, an efficient one is the measurement of a point machine’s gap; i.e., the real gap between the lock bar notch and the edge of the lock hammer, as shown in Figure 1. This gap may be used as an indirect measurement of gap width between switch point and stock rail. The former can be easily converted to the latter, and the gap width between switch point and stock rail can therefore be calculated. Following this idea, some researchers have devoted a large amount of effort in this area. Franke [5] presented a device that uses inductive proximity sensors, and Hager [6] used a transformer with two coils to detect the point machine gap. Melich proposed a measurement with a magnetic field produced by two magnets and two Hall effect sensors [7]. This approach, however, has two problems. The first is that the analog signal from the sensor has to be calibrated in order to give a measure of the gap in millimeters; another problem is that the wires or the sensors themselves may be broken during maintenance tasks. Melich also used an image binarization and morphological filtering to measure the point machine gap. Lou proposed a point machine gap measurement method [8] which integrates median filtering methods and edge image binarization to extract gap edges and then calculate the point machine gap.

The above studies improve the precision of gap measurement. They utilize traditional image processing technologies, such as image denoising and image binarization. However, there are three problems requiring advancements in the processing of gap images. The first is that an unsuitable image denoising method may blur edges in gap images, which may set up barriers for the following edge detection and gap width calculation. The second one is that a local intensity variation often appears in gap images due to uneven illumination—this may lead to missing a gap edge by simply adopting global thresholds. Finally, in edge detection and gap measurement, an unsuitable structure element may be useless for reducing spectacle spot caused by metal surface reflection.

To tackle these problems, we propose an improved gap measurement algorithm which integrates an adaptive wavelet threshold, local threshold in image binarization, and line structure element in mathematical morphology. An adaptive wavelet threshold can obtain the optimal threshold in wavelet-based image denoising. This will greatly suppress Gaussian noise imposed on the images and keep the unblurred gap edges; a local threshold rather than a global one may help to search gap edges under uneven illumination; and line structure element in mathematical morphology may greatly reduce the spectacle spot due to metal surface reflection. A subject and object evaluation is presented to validate the effectiveness of the proposed method.

The rest of this paper is organized as follows. Section 2 provides an overview of a gap measurement system for point machines. Section 3 elaborates the proposed method, including adaptive threshold-based wavelet denoising, local threshold-based image binarization, and line structure element-based mathematical morphology. Section 4 presents a subject and object evaluation of the proposed method. Finally, the paper is concluded in Section 5.

## 2. System Overview

**Figure 2:**
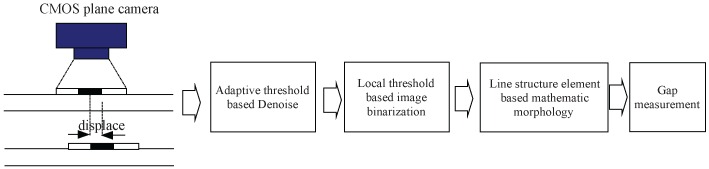
Schematic diagram of the gap measurement.

Figure 2 illustrates a schematic diagram of the gap measurement system. The most critical factor determining the camera image quality is the type of the image sensor. There are two basic types of image sensor: CCD (charge-coupled device) and CMOS (complementary metal oxide semiconductor). Traditionally, CCD sensors have been thought to produce better-looking images with less visual noise and distortion, but they draw more power and provide slower data-throughput speed. CMOS is increasingly used in today’s cameras, allowing users to shoot high-resolution video and apply complex imaging effects with ease [9]. In this gap measurement system, a CMOS plane camera with resolution 750 × 480 and focus length 3.6 mm is adopted. In Figure 2, a camera with CMOS plane is firstly used to capture two images of black and white stripes pasted on a lock bar. Among these two stripes, one is used as a reference gap tag. Therefore, the gap width is indirectly measured by the displacement between these two stripes. The gap measurement system transfers these images to the image processing unit, and then an adaptive threshold-based wavelet denoising is carried out to obtain the optimal threshold and suppress Gaussian white noise. Second, a local threshold-based image binarization is used to decrease the chance of edge missing due to uneven illumination. Afterward, a line structure element-based mathematical morphology is applied to reduce the speckle spot caused by material surface reflection. Finally, the gap measurement is gained by calculating the displacement between edges of corresponding two stripes.

## 3. Methodology

### 3.1. Adaptive Wavelet Threshold-Based Noise Removal

Usually, a CMOS detector is characterized by a linear model. Then, for the ${\left(ij\right)}^{th}$ detector in the CMOS plane, the measured readout signal $g_{ij}$ can be expressed as [10]:

(1) $$g_{ij}=a_{ij}\cdot f_{ij}+\epsilon_{ij}$$

where $a_{ij}\left(t\right)$ are the gain and $\epsilon_{ij}$ the added noise of the ${\left(ij\right)}^{th}$ detector, and $f_{ij}\left(t\right)$ is the real incident radiation collected by the respective detector. Because the gain is not obvious in the captured images, we omit it in this work. $\epsilon_{ij}$ are independent and identically distributed (*iid*) as normal $N(0,\sigma^{2})$ and independent of $\left\{f_{ij}\right\}$.

The objective of denoising is to remove the noise, or “denoise” $g_{i,j}$, and to obtain an estimate ${\hat{f}}_{ij}$ of $f_{ij}$ while minimizing the mean squared error (MSE),

(2) $$MSE\left(\hat{f}\right)=\frac{1}{MN}\sum_{i,j}{\left({\hat{f}}_{ij}-f_{ij}\right)}^{2}$$

where *M* and *N* are the image sizes.

It should be noted that the edges in the image are not blurred by the denoising operation, because these edges always carry important information for the following gap measurement. On one hand, the traditional filter templates (such as “average”) can remove the noise. However, on the other hand, it also blurs the edge, which will set up an obstacle for the following edge detection. Therefore, we here use wavelet-based image denoise technology. Image denoising based on the wavelet transform is mainly completed by wavelet thresholding in wavelet domain [11,12]. The processing of image denoising in wavelet domain can be considered as an optimal estimation to the input image with noise data using the threshold.

Let $g={\left\{g_{ij}\right\}}_{i,j},f={\left\{f_{ij}\right\}}_{i,j}$, and $\varepsilon ={\left\{\epsilon_{ij}\right\}}_{i,j}$. Here we borrow the idea from [13] to use Bayesian to find the best soft-threshold rules under the Gaussian assumption (i.e., $X∼N(0,\sigma_{X}^{2})$), which can efficiently utilize the image prior knowledge. Formally, the objective of image denoising is to find a threshold *T* which minimizes the Bayes risk,

(3) $$risk\left(T\right)=E{\left(\hat{X}-X\right)}^{2}=E_{X}E_{Y|X}{\left(\hat{X}-X\right)}^{2}$$

where $\hat{X}=\rho_{T}\left(Y\right)$, Y=Wg,X=Wf,V=Wε, and *W* is the two-dimensional dyadic orthogonal wavelet transform operator. $Y|X∼N(x,\sigma^{2})$. $\rho_{T}\left(\right)$ is the threshold function. For soft-threshold function, $\rho_{T}\left(x\right)=sgn\left(x\right)\cdot \max\left(|x|-T,0\right)$ takes the argument and shrinks it toward zero by the threshold *T*.

Equation (3) can be reformulated as follows:

(4) $$\begin{array}{ccc} risk(T) & = & E_{X}E_{Y|X}{\left(\hat{X}-X\right)}^{2} \end{array}$$

(5) $$\begin{array}{ccc} & = & \int_{-\infty }^{\infty }\int_{-\infty }^{\infty }{\left(\rho_{T}\left(y\right)-x\right)}^{2}p\left(y|x\right)p\left(x\right)dydx \end{array}$$

(6) $$\begin{array}{ccc} & = & \sigma^{2}+\sqrt{\frac{2}{\pi }}\left(T^{2}+\sigma^{2}-\sigma_{X}^{2}\right)\int_{\frac{T}{\sigma_{y}}}^{\infty }\exp\left(-\frac{T^{2}}{2}\right) \end{array}$$

(7) $$\begin{array}{ccc} & & -\sqrt{\frac{2}{\pi }}T\sigma \sigma_{y}\exp\left(-\frac{T^{2}}{2\sigma_{y}^{2}}\right) \end{array}$$

where $\sigma_{y}^{2}=\sigma^{2}+\sigma_{X}^{2}$. In contrast to [13], the $\sigma_{X}$ in the captured point machine gap images is almost same, because only the gap location moved slowly, and the background is almost unchanged. Therefore, by using the wavelet operation *W* and the Bayes-based calculation of optimal threshold, the adaptive wavelet threshold $T^{*}$ is

(8) $$T^{*}\left(\sigma \right)=\arg\min_{T}risk\left(T\right)$$

**Figure 3:**
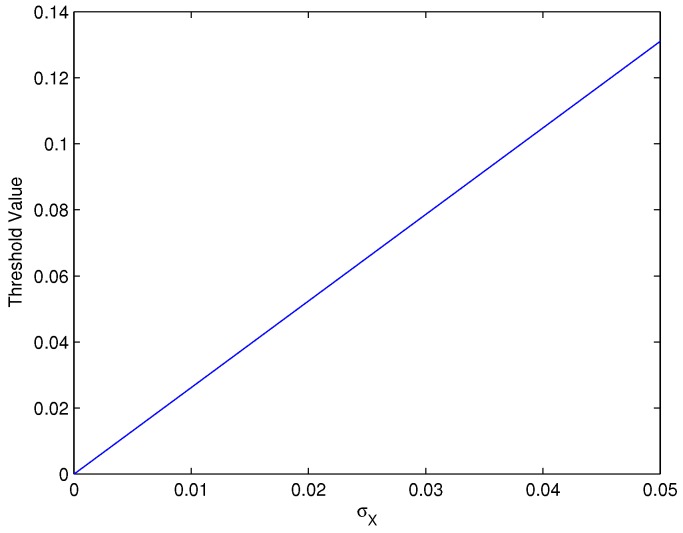
Thresholding for different Gaussian noise variance.

The value of $T^{*}\left(\sigma \right)$ is found numerically for different values of $\sigma^{2}$ under the $\sigma_{X}=0.3816$, as shown in Figure 3. From it, we can set the optimal threshold for image denoising.

**Figure 4:**
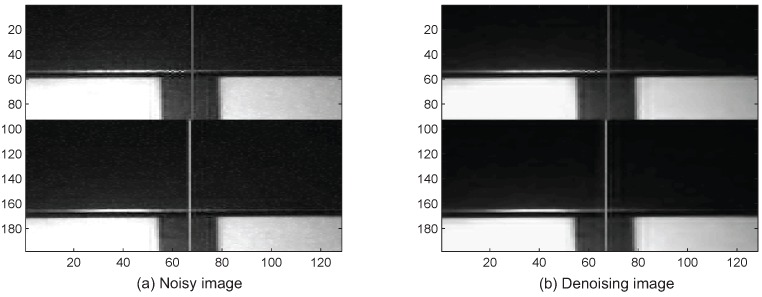
Noisy and denoising image.

Figure 4 presents the noisy image with $\sigma^{2}=0.02$ and the denoising one by applying the generated optimal threshold 0.052. Considering that it is impossible to obtain true noise-free images, we have to carefully select some with low noise as noise-free images. The simulated noisy images are obtained by adding different levels of noise.

### 3.2. Local Threshold-Based Image Binarization

**Figure 5:**
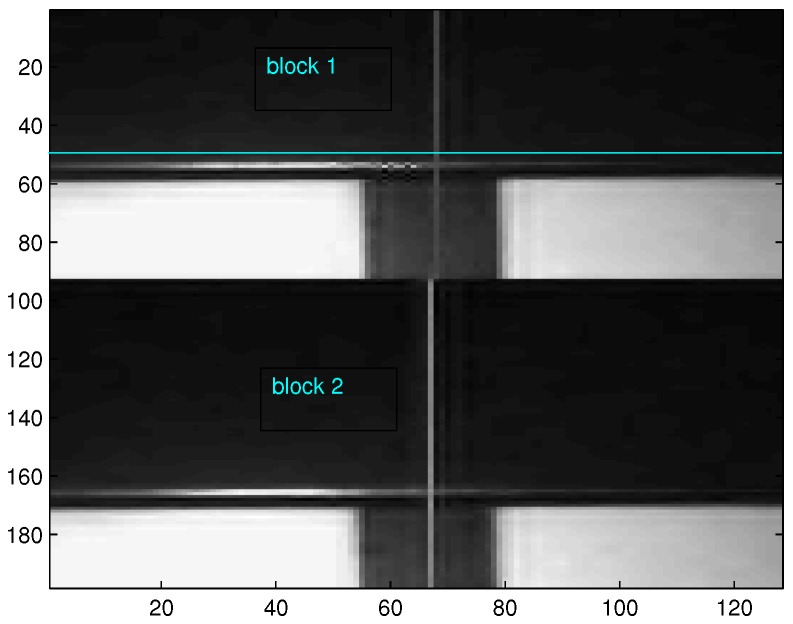
Two blocks in the divided input image.

**Figure 6:**
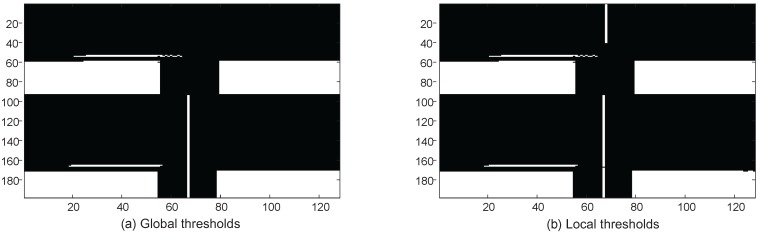
Comparison between global and local thresholds.

In order to perform edge detection, a grey image should be transformed into a binary one. During this process, an optimal threshold plays a key role. There are well-known global and local threshold algorithms [14,15]. Compared with the global thresholding algorithms, local ones are superior in terms of selecting threshold values according to local intensity variation. Specifically in point machine gap images, the local intensity variation often appears due to uneven illumination. In order to solve this problem, an adaptive local binarization method, as shown in Algorithm 1, is proposed by dividing the gap images into several parts and then applying Otsu’s method [14] to each part. Otsu’s method chooses the threshold to minimize the intraclass variance of the black and white pixels to form a binary image. Figure 5 illustrates that two parts are divided in a point machine gap image, in which each part uses Otsu’s method to generate the binary images. Figure 6 gives the comparison between the global threshold and the local one. From this figure, we can see that if a global threshold is used, a gap calibration line (i.e., the vertical white line in the upper image) is lost due to weak illumination. Otherwise, a local threshold can be used to overcome the uneven illumination and capture the gap calibration line, which is shown in Figure 6b.

**Algorithm 1:** Adaptive local image binarization method.
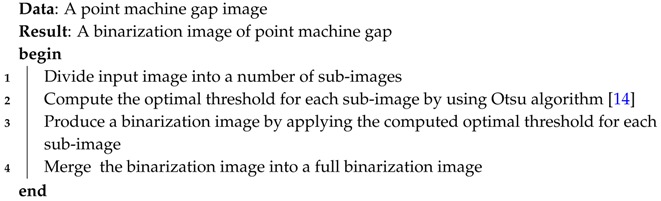


### 3.3. Line Structure Element-Based Mathematical Morphology

Mathematical morphology is a set-theoretic method of image analysis providing a quantitative description of geometrical structures. The fundamental idea of mathematic morphological edge detection is that an edge which satisfies the shape of a structure element is extracted according to the morphological edge detection operator [16]. By utilizing suitable geometrical structures, the methods of edge detection based on the morphology transform are capable of obtaining clear edges and suppressing the noise—especially impulse noise, such as speckles in the case of a reflective metal surface.

**Figure 7:**
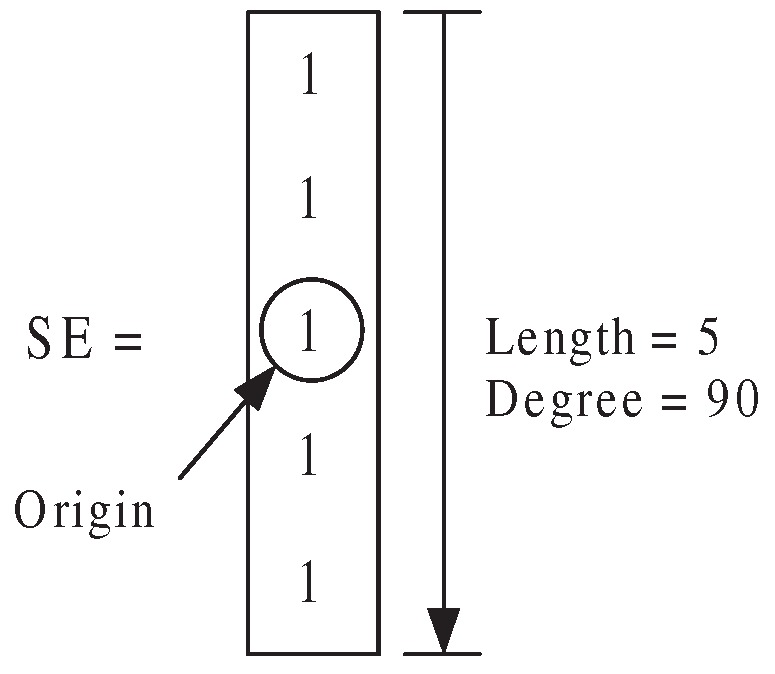
Structure element.

Considering the special shape of point machine gaps, we here apply a line structure element, as shown in Figure 7. This creates a flat, linear structuring element, where “Length” specifies the length, and is approximately the distance between the centers of the structuring element members at opposite ends of the line. “Degree” specifies the angle (in degrees) of the line, as measured in a counterclockwise direction from the horizontal axis.

**Figure 8:**
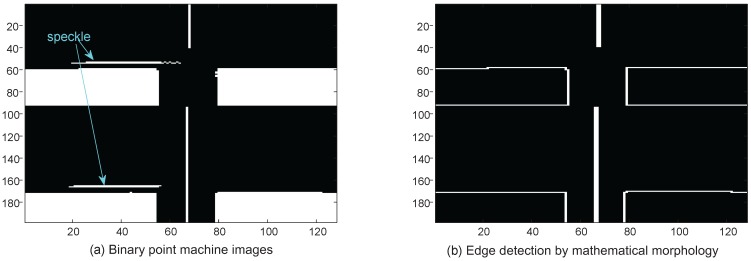
Mathematical morphology-based edge detection.

Figure 8 presents the edge detection by performing morphological opening on the binary image with the proposed line structuring element. The morphological opening operation is an erosion followed by a dilation, using the same line structuring element for both operations. Figure 8a illustrates the binary point machine images, and Figure 8b the edge detection by mathematical morphology. Using the aforementioned line structuring element shown in Figure 7, we can see that some speckles caused by the reflective metal surface are removed, and the point machine gap edges are extracted clearly.

The gap edges can be easily calculated by multiplying the number of pixels between gap edges by the resolution between two pixels.

## 4. Experimental Results

### 4.1. Subjective Evaluation

The proposed method is tested on a number of point machine gap images recorded in a railway corporation in China, including low level noise images and noisy images. The former can be regarded as noise-free images due to their high quality. The latter may result from electromagnetic interference or communication channels.

**Figure 9:**
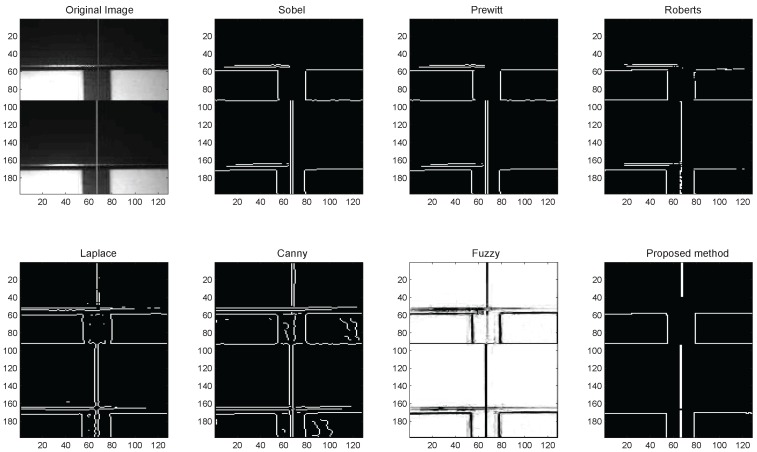
Comparison between conventional and the proposed methods for edge detection in noise-free gap images.

In noise-free images, Figure 9 gives the comparison of the proposed method with traditional edge detection methods, such as Sobel [17], Prewitt [18], Roberts [19], Laplace [20], Canny [21], and fuzzy logic [22] based techniques. For Sobel, Prewitt, Roberts, Laplace, and Canny based edge detections, the noise-free image is convolved with their kernels to approximate the derivatives in horizontal and vertical change. Heuristic thresholds are used, which are based on RMS (root mean square) estimates of the mean of the magnitude squared image. The fuzzy method uses membership functions to define the degree to which a pixel belongs to an edge or a uniform region. Similar to [22], we define a fuzzy inference system with a zero-mean Gaussian membership function for inputs and triangular membership functions for outputs. Then, some fuzzy inference rules were added to make a pixel white if it belongs to a uniform region, and otherwise, it was made black. The result of the edge detection is obtained by defuzzification.

We can see that the proposed method successfully detects all types of edges and suppresses the speckle spot caused by reflective metal surfaces. However, the traditional method may generate false edges or lose true ones, which will create obstacles for the following point machine gap measurement.

**Figure 10:**
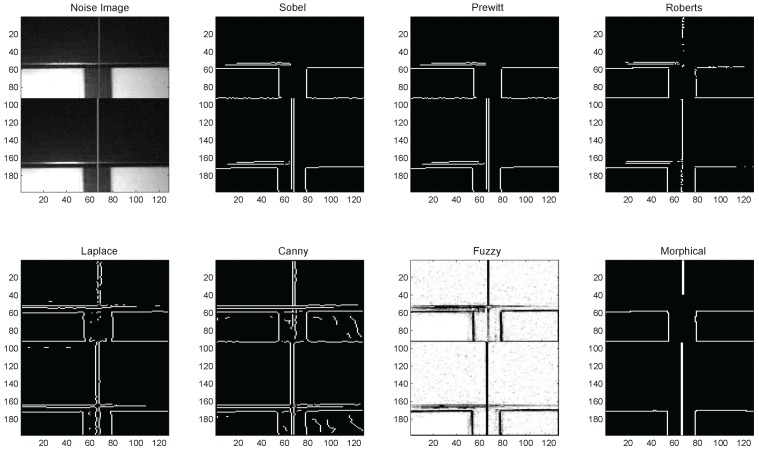
Comparison between conventional and the proposed methods for edge detection in noisy gap images.

At the same time, the proposed method has the advantage of detecting point machine edges in noisy images without blurring edge pixels. This is verified by detecting edges in point machine images with Gaussian white noise with variance 0.02. Figure 10 illustrates the comparison between the proposed method and the aforementioned conventional edge detection algorithms, in which heuristic thresholds are used. These heuristic thresholds are based on RMS estimate of noise mean of the magnitude squared image, and are roughly proportional to the SNR (signal-to-noise ratio). From the experimental results, it is clear that the proposed method detects very few false edge pixels compared to other reported edge detection techniques.

### 4.2. Objective Evaluation

Measures for evaluating the performance of edge detectors have been formulated by Abdou and Pratt [23] and DeMicheli, Caprile, Ottonello, and Torre [24]. The criteria to consider in evaluating the performance of an edge detector include

1. Probability of false edges;
2. Probability of missing edges;
3. Error in estimation of the edge angle;
4. Mean square distance of the edge estimate from the true edge;
5. Tolerance to distorted edges and other features such as corners and junctions.

The first two criteria concern the performance of an algorithm as a detector of edges. The second two criteria concern the performance of an algorithm as an estimator of the edge location and orientation. The last criterion concerns the tolerance of the edge algorithm to edges that depart from the ideal model used to formulate the algorithm. We here use a set of direct measurements, such as the number of correctly detected edge pixels (called true positive), the number of pixels erroneously classified as edge pixels (called false positive), and the amount of edge pixels that were not classified as edge pixels (called missed).

**Figure 11:**
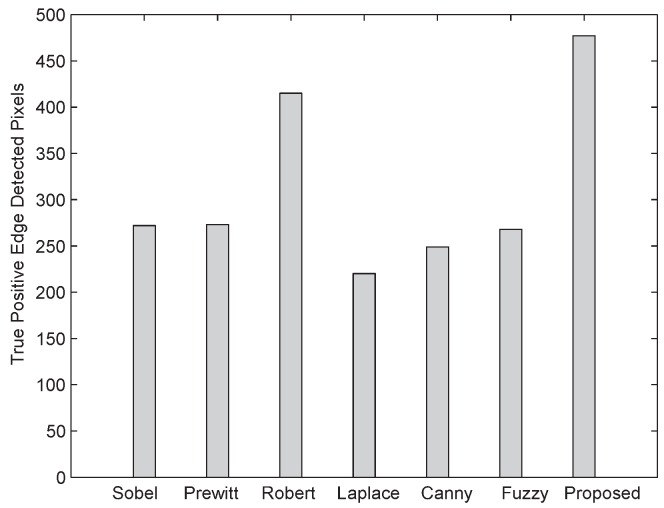
Comparison of true positive edge detected pixels between the proposed algorithm and other conventional ones.

**Figure 12:**
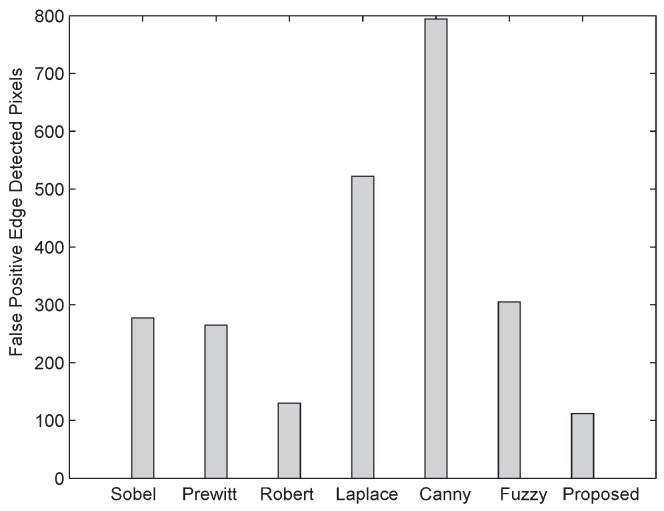
Comparison of false positive edge detected pixels between the proposed algorithm and other conventional ones.

**Figure 13:**
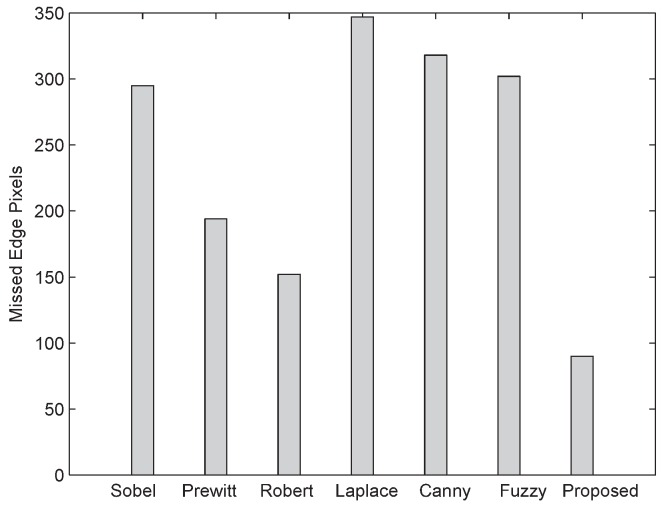
Comparison of missed edge pixels between the proposed algorithm and other conventional ones.

The number of positive edge pixels, false edge pixels, and missed edge pixels detected by different reported edge detection techniques are shown in Figure 11, Figure 12 and Figure 13, respectively. From Figure 12, it is evident that the proposed algorithm when subject to a noisy image of 512 × 512 size and 25 dB noise level has detected 102 false edge pixels, while other edge detection techniques—For instance, Sobel [17], Prewitt [18], Laplace [20], Roberts [19], Canny [21], and fuzzy logic [22] based techniques—after fine tuning, the Canny method gives more false edge pixels. As shown in Figure 11 and Figure 13, the proposed algorithm gives more positive edge pixels and less missed edge pixels than other edge detection techniques.

In this work, the performance is next compared based on the parameters mean square error (MSE) and peak signal-to-noise ratio (PSNR). MSE indicates the average difference of the pixels throughout the image. A higher MSE indicates a greater difference between the original and processed image. Nevertheless, it is necessary to be very careful with the edges. The formula for the MSE calculation is given as:

(9) $$MSE=\frac{1}{M*N}\sum_{i}\sum_{j}{\left(X_{ij}-V_{ij}\right)}^{2}$$

where *M* and *N* are the size of the image, *X* is the processed image, and *V* is the original image. Peak Signal-to-Noise Ratio (PSNR) is formulated as

(10) $$PSNR=10*\mathrm{lg}(\frac{M*N*255^{2}}{\sum_{i}\sum_{j}{\left(X_{ij}-V_{ij}\right)}^{2}})$$

which is used for quantitative comparison of different methods.

As shown in Table 1, the proposed method has the smallest MSE and the largest PSNR, so it has the best performance.

Finally, the performance is compared based on the parameters $SD_{k}$ [25]. $SD_{k}$ has better theoretical properties and is sensitive to both False Positive (FP) and False Negative (FN). When k=1 or k=2 we obtain the average symmetric surface distance or root mean square symmetric surface distance, respectively, as introduced by Heimann et al. [26]. The formula for $SD_{k}$ calculation is given as:

(11) $$SD_{k}\left(E_{gt},E_{c}\right)=\frac{{\left(\Sigma_{p\in E_{c}}d^{k}\left(p,E_{gt}\right)+\Sigma_{p\in E_{gt}}d^{k}\left(p,E_{c}\right)\right)}^{\frac{1}{k}}}{\left(\right|E_{c}\cup E_{gt}{\left|\right)}^{\frac{1}{k}}}$$

which is used for quantitative comparison of different methods. In Equation (11), $E_{gt}$ is perfect solution (ground truth) to the edge detection problem, $E_{c}$ a candidate edge image, *p* a pixel, $d(p,E_{gt})$ the distance from *p* to the closest point in $E_{gt}$, |·| cardinality of set.

From Table 2, we can see that the proposed method has the smallest $SD_{1}$ and $SD_{2}$. Therefore it gains the best performance.

## 5. Conclusions

This work proposes an image-based point machine gap measurement method. The proposed method can obtain an optimal denoising by using an adaptive wavelet-based threshold while keeping gap image edges unblurred, which plays an important role in precise gap measure. It also reduces edge missing by using local rather than global threshold in image binarization. A line structure element in mathematic morphology helps to reduce the speckle spot caused by metal surface reflection. Point machine gap images from a railway corporation have been used to validate the effectiveness of the proposed method via both subjective and objective evaluations. A comparison experiment shows that the proposed method outperforms the traditional ones. In image binarization, a more intelligent method is needed in order to obtain the optimal block division and local threshold; this is left for future work.

## Figures and Tables

**Table 1:** Comparison of mean square error (MSE) and peak signal-to-noise ratio (PSNR) between the proposed algorithm and other conventional ones.

	Sobel	Prewitt	Robert	Laplace	Canny	Proposed Algorithm
MSE	0.1812	0.1814	0.1347	0.2204	0.2429	0.1193
PSNR	55.5492	55.5444	56.8371	54.6987	54.2765	57.3644

**Table 2:** Comparison of $SD_{1}$ and $SD_{2}$ between the proposed algorithm and other conventional ones.

	Sobel	Prewitt	Robert	Laplace	Canny	Proposed Algorithm
k=1	1.3509	1.3450	1.2246	1.8774	1.9826	0.1563
k=2	37.5285	37.2919	34.5551	58.8145	64.6885	3.6443

## References

[1] National Railway Administration of the People’s Repubilc of China (2016). Railway Statistical Bulletin in 2015. http://www.nra.gov.cn/fwyd/zlzx/hytj/.

[2] Morant A., Larsson-Kraik P.O., Kumar U. (2016). Data-driven model for maintenance decision support—A case study of railway signalling systems. Proc. Inst. Mech. Eng. Part F J. Rail Rapid Transit.

[3] Budai-Balke G. (2009). Operations Research Models for Scheduling Railway Infrastructure Maintenance. Ph.D. Thesis.

[4] Xu T., Wang H., Yuan T., Zhou M.C. (2016). BDD-based synthesis of fail-safe supervisory controllers for safety-critical discrete event systems. IEEE Trans. Intell. Transp. Syst..

[5] Franke R.C. (1999). Railway switch machine point detection system. Patent.

[6] Hager M.A., Towey M.F. (2001). Contactless point detection system for railroad switch. Patent.

[7] Melich R.M. (2015). Position estimation and gap measurement of a point machine using an electronic device with embedded artificial vision framework. Masters’ Thesis.

[8] Lou S. (2013). Study on Switch Machines Gap Detection System Based on Video Image Recognition Technology. Masters’ Thesis.

[9] CMOS Is Winning the Camera Sensor Battle. http://www.techhive.com/article/246931/cmos_is_winning_the_camera_sensor_battle_and_heres_why.html?page=1/2.

[10] Shahram M., Sobhan R., Mehdi Nasiri S. Fixed pattern noise reduction method in CCD sensors for LEO satellite applications. Proceedings of the 2011 11th International Conference on Telecommunications (ConTEL).

[11] Mustafa N., Li J.P., Kh S.A., Giess M. Medical image de-noising schemes using wavelet threshold techniques with various noises. Proceedings of the International Computer Conference on Wavelet Active Media Technology and Information Processing.

[12] Chen Y., Han C. (2005). Adaptive wavelet threshold for image denoising. Electron. Lett..

[13] Chang S.G., Yu B., Vetterli M. (2000). Adaptive wavelet thresholding for image denoising and compression. IEEE Trans. Image Process..

[14] Otsu N. (1979). A threshold selection method from gray-level histograms. IEEE Trans. Syst. Man Cybern..

[15] Liu Y., Srihari S.N. (1979). Document image binarization based on texture features. IEEE Trans. Pattern Anal. Mach. Intell..

[16] Serra J. (1982). Image Analysis and Mathematical Morphology.

[17] Zhang J.Y., Chen Y., Huang X.X. (2009). Edge detection of images based on improved sobel operator and genetic algorithms. IEEE Int. Conf. Image Anal. Signal Process..

[18] Yang L., Zhao D., Wu X., Li H. (2011). An improved Prewitt algorithm for edge detection based on noised image. IEEE Int. Congr. Image Signal Process..

[19] Rosenfeld A. (1981). The Max Roberts Operator is a Hueckel-Type Edge Detector. IEEE Trans. Pattern Anal. Mach. Intell..

[20] Vliet L.J.V., Young I.T., Beckers G.L. (1989). A nonlinear laplace operator as edge detector in noisy images. Comput. Vision Graph. Image Process..

[21] Canny J.A. (1986). Computational Approach to Edge Detection. IEEE Trans. Pattern Anal. Mach. Intell..

[22] Haq I., Anwar S., Shah K., Khan M.T., Shah S.A. (2015). Fuzzy Logic Based Edge Detection in Smooth and Noisy Clinical Images. PLoS ONE.

[23] Abdou I.E., Pratt W.K. (1979). Quantitative design and evaluation of enhancement/thresholding edge detectors. Proc. IEEE.

[24] Micheli E.D., Caprile B., Ottonello P., Torre V. (1989). Localization and Noise in Edge Detection. IEEE Trans. Pattern Anal. Mach. Intell..

[25] Lopez-Molina C., Baets B.D., Bustince H. (2013). Quantitative error measures for edge detection. Pattern Recognit..

[26] Heimann T., Van G.B., Styner M.A., Arzhaeva Y., Aurich V., Bauer C., Beck A., Becker C., Beichel R., Bekes G. (2009). Comparison and evaluation of methods for liver segmentation from CT datasets. IEEE Trans. Med. Imaging.

